# Evaluation of fan-beam kilovoltage computed tomography image quality on a novel biological-guided radiotherapy platform

**DOI:** 10.1016/j.phro.2023.100438

**Published:** 2023-04-10

**Authors:** Tingliang Zhuang, Grant Gibbard, Xinhui Duan, Jun Tan, Yang Park, Mu-Han Lin, Zhihui Sun, Oluwaseyi M. Oderinde, Weiguo Lu, Robert Reynolds, Andrew Godley, Arnold Pompos, Tu Dan, Aurelie Garant, Puneeth Iyengar, Robert Timmerman, Steve Jiang, Bin Cai

**Affiliations:** a Department of Radiation Oncology, University of Texas- Southwestern Medical Center, Dallas, USA; b Department of Radiology, University of Texas- Southwestern Medical Center, Dallas, USA; c RefleXion Medical, Inc, Hayward, CA, USA

**Keywords:** PET/CT Linac, kV fan-beam CT, CT simulator, Image quality

## Abstract

**Background and Purpose:**

A recently developed biology-guided radiotherapy platform, equipped with positron emission tomography (PET) and computed tomography (CT), provides both anatomical and functional image guidance for radiotherapy. This study aimed to characterize performance of the kilovoltage CT (kVCT) system on this platform using standard quality metrics measured on phantom and patient images, using CT simulator images as reference.

**Materials and Methods:**

Image quality metrics, including spatial resolution/modular transfer function (MTF), slice sensitivity profile (SSP), noise performance and image uniformity, contrast-noise ratio (CNR) and low-contrast resolution, geometric accuracy, and CT number (HU) accuracy, were evaluated on phantom images. Patient images were evaluated mainly qualitatively.

**Results:**

On phantom images the MTF_10%_ is about 0.68 lp/mm for kVCT in PET/CT Linac. The SSP agreed with nominal slice thickness within 0.7 mm. The diameter of the smallest visible target (1% contrast) is about 5 mm using medium dose mode. The image uniformity is within 2.0 HU. The geometric accuracy tests passed within 0.5 mm. Relative to CT simulator images, the noise is generally higher and the CNR is lower in PET/CT Linac kVCT images. The CT number accuracy is comparable between the two systems with maximum deviation from the phantom manufacturer range within 25 HU. On patient images, higher spatial resolution and image noise are observed on PET/CT Linac kVCT images.

**Conclusions:**

Major image quality metrics of the PET/CT Linac kVCT were within vendor-recommended tolerances. Better spatial resolution but higher noise and better/comparable low contrast visibility were observed as compared to a CT simulator when images were acquired with clinical protocols.

## Highlights

• Described kilovoltage computed tomography (kVCT) on a novel biology-guided radiotherapy linac.
• Characterized imaging performance of the on-board kVCT based upon national standard.
• Compared image quality of the on-board kVCT to CT simulator on phantom and patients.
• kVCT system on biology-guided linac can potentially improve soft tissue image guidance accuracy.

## 1 Introduction

Image guidance has been extensively used in radiotherapy for cancer treatment with improved accuracy of patient positioning and target localization. On-board X-ray imaging system on the L-shaped gantry system implemented by major vendors can provide three-dimensional cone-beam CT (CBCT) images for target localization [1], [2]. However, cone-beam data acquisition geometry introduced several artifacts into the reconstructed images, including scatter induced shading and streaking artifacts as well as cone-beam artifacts. Despite many efforts devoted to mitigating the scatter induced artifacts (see [3] and references therein), the soft-tissue contrast in CBCT images remains inferior to that of images acquired with the diagnostic CT or simulation CT system using a fan-beam geometry [4], [5]. In-room CT-on-rail system can provide better image quality, however, mechanical transportation/rotation and repositioning of the patient is required after image acquisition [6], [7].

A recently designed radiotherapy platform has incorporated an on-board kV fan-beam CT (kVCT) and Positron Emission Tomography (PET) system into a ring-shaped gantry that can provide both anatomical and functional images [8]. A few studies on this novel radiotherapy platform have been reported regarding the initial machine commissioning [9], the characteristics of the PET imaging system performance [10], treatment planning experiences [11], [12] as well as a detailed clinical workflow for using this system for biology-guided radiotherapy [13]. However, the imaging performance of the kVCT system has not been investigated. The aim of this study was to comprehensively evaluate the imaging performance of the kVCT system on this PET/CT linac using both phantom and patient images. We hypothesize that the PET/CT linac kVCT system may achieve similar quality as a CT simulator, which may improve accuracy of soft-tissue based image guidance as well as organ delineation for potential adaptive radiotherapy.

## 2 Material and methods

### 2.1 Overview of the kVCT IGRT system on the PET/CT linac (RefleXion® X1 system)

The kVCT system on RefleXion X1(RFX1) utilizes helical acquisition in clinical mode with a fixed x-ray tube potential at 120 kVp and adjustable mAs and pitch. A cross-section view of the major components of the RFX1 system is shown in Fig. S1 in the supplement material and in Ref. [8].

### 2.2 Scanning protocols and image quality evaluation

This study used images of a CT phantom (Catphan604, The Phantom Laboratory, New York, NY) and patients to evaluate the kVCT imaging performance. The image quality metrics including in-plane spatial resolution using both the bar pattern and the modular transfer function (MTF), cross-plane resolution using slice sensitivity profile (SSP), image uniformity and noise, contrast-noise ratio (CNR), low contrast visibility, CT number accuracy, and geometry accuracy were measured on the Catphan images. An in-house Matlab (The MathWorks Inc., Natick, MA) code was developed to extract these image quality metrics semi-automatically.

Catphan images acquired on RFX1 kVCT were directly compared to those acquired from a Philips Brilliance Big Bore CT simulator (Philips Medical Systems, Cleveland, OH) in our department. The Catphan604 has various modules with different inserts with reference density and geometry for quantitative image quality assessment (see supplementary material for details). Clinical protocol parameters are listed in Table 1 for both phantom and patient image acquisitions. Note that the imaging dose (*CTDI_vol_*) are different between the RFX1 kVCT acquisitions and the CT simulator.

**Table 1:** The data acquisition and image reconstruction parameters for the clinical protocols in RFX1 kVCT and Philips CT simulator for phantom and patients. BLM = Body/Low dose/Medium pitch; BMM = Body/Medium dose/Medium pitch; BHM = Body/High dose/Medium pitch; BHS = Body/High dose/Slow pitch.

	**RFX1**				**CT simulator**		
**Clinical protocol**	**BLM**	**BMM**	**BHM**	**BHS**	**Neck**	**SBRT abdomen**	**SBRT lung**
**X-ray collimation**	16x1.25				16x0.75	16x1.5	16x1.5
**kVp**	120				120		
**Effective mAs/mA**	200/100	300/150	600/300	600/133	150/163	303/328	334/362
**Tube current modulation**	N/A				DoseRight Index 21		
***CTDI_vol_*(mGy)**	16.3	24.7	49.0	48.9	9.9*	18.0*	19.7*
**Pitch**	0.5		0.222		0.813		
**Rotation time (s)**	1				0.75		
**FOV(mm)**	500				500	700	700
**Slice Thickness (mm)**	1.25				3	2	2
**Reconstruction algorithm**	FDK				iDose^4^		
**Kernel**	Standard				B/C/YA†	C	

* The computed tomography dose index-volume (CTDI_vol_) values listed here are imaging dose specifically for the simulation CT scan of the phantom and the two example patients shown in this paper later.

† B: Smoothed, but sharper and noisier than A. Recommended for CTA, routine abdomen, and pelvis; C: Sharper, creates relatively low-noise images. Recommended for CTA, routine abdomen, and pelvis to get slightly higher sharpness than with Filter B; YA: Sharper and noisier. Recommended for reconstruction of sinuses, facial bones, dental, etc. The thinner the axial slices the sharper the axial, 2D and 3D images. (cited from Philips CT simulator user manual).

#### 2.2.1 Image quality metrics

##### 2.2.1.1 In-plane spatial resolution

The qualitative assessment on the in-plane spatial resolution was performed by visual inspection of the bar pattern in the CTP732 module. A quantitative metric, the modular transfer function (MTF) was computed by using the circular edge method [14], [15] to evaluate the spatial resolution (see supplementary material for details). The spatial resolution at MTF that equals 10% is roughly corresponding to the “vanishing resolution” beyond which the pattern of alternating high contrast bars is not visually differentiable.

##### 2.2.1.2 Cross-plane spatial resolution (slice sensitivity profile)

**Fig. 1:**
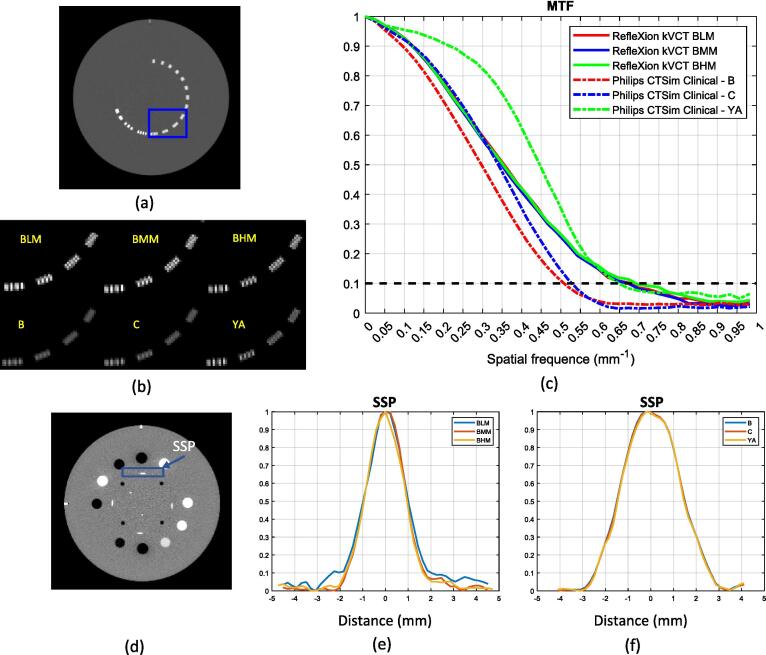
(a) Axial image of the bar pattern. (b) The zoomed-in image of the rectangular region in (a) for the three RFX1 kVCT acquisitions and three CT simulator reconstructions. Display window is the same for all the bar pattern images. (c) The MTF. (d) Axial image of the HU module that consists reconstructed slanted wire (blue rectangular region) for SSP measurement. (e) and (f) SSP for RFX1 kVCT and CT simulator averaged over five consecutive slices. (For interpretation of the references to colour in this figure legend, the reader is referred to the web version of this article.)

The slice sensitivity profile (SSP) was measured using the slanted wire method (see section 5.1.2 in Chapter 5 of [16]). The intensities along a horizontal line centered on the slanted wire on an axial image shown in Fig. 1(d) were extracted first. The SSP was then obtained by plotting the measured intensity with the horizontal axis scaled by a factor of $tan(23^{o})$. The slice thickness was obtained as the full width at half maximum (FWHM) on the SSP averaged over five consecutive slices.

##### 2.2.1.3 Image uniformity and noise

**Fig. 2:**
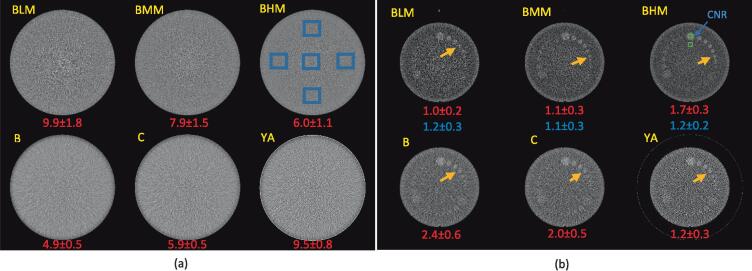
Comparisons of axial images of (a) uniformity module and (b) low contrast inserts module for the two imaging systems. (a) The five blue rectangular region on RFX1 kVCT image with BHM mode indicates ROIs for noise and uniformity calculation. The value of the noise level is listed at the bottom of each image. (b) The two green rectangular region on RFX1 kVCT image with BHM mode indicates ROIs for CNR calculation. The value of CNR is listed in red at the bottom of each image. The scaled CNR for RFX1 kVCT images are listed in blue. (For interpretation of the references to colour in this figure legend, the reader is referred to the web version of this article.)

The uniformity was computed as the maximum absolute difference of the mean value between the center region-of-interest (ROI, 20x20 pixels) and the peripheral ROIs as shown in Fig. 2(a). The image noise σ is quantified by the standard deviation of the CT number within each ROI. Taking into account the differences in imaging dose (${\mathrm{CTDI}}_{\mathrm{vol}}$) and slice thickness (ST), a scaled noise metric from the standard deviation measured on the RFX1 CT images ${\hat{\sigma }}_{\mathrm{RefCT}}$ were also computed using Eq. (1) to compare the noise performance between the two imaging systems.

(1) $${\hat{\sigma }}_{\mathrm{RefCT}}=\sigma_{\mathrm{RefCT}}\sqrt{\frac{{\left({\mathrm{CTDI}}_{\mathrm{vol}}*ST\right)}_{\mathrm{RefCT}}}{{{(CTDI}_{\mathrm{vol}}*ST)}_{\mathrm{SimX}}}}$$

##### 2.2.1.4 Contrast-noise ratio (CNR) and low contrast visibility

The CNR was computed with the following formula:

(2) $$\mathrm{CNR}=\frac{|{\overset{¯}{\mathrm{HU}}}_{A}-{\overset{¯}{\mathrm{HU}}}_{B}|}{\sigma_{B}}$$

where ${\overset{¯}{\mathrm{HU}}}_{A/B}$ is the mean pixel values in the square ROI_A/B_, and $\sigma_{B}$ is the standard deviation of the pixel values in ROI_B_.

The ROI_A/B_ (size 6x6 pixels) were shown in Fig. 2(b). The CNR was averaged over the nearest five slices. A scaled contrast-noise ratio with the noise replaced by the scaled noise was computed by using $\hat{\mathrm{CNR}}=CNR\frac{\sigma_{B}}{\hat{\sigma_{B}}}$.

##### 2.2.1.5 CT number accuracy

**Fig. 3:**
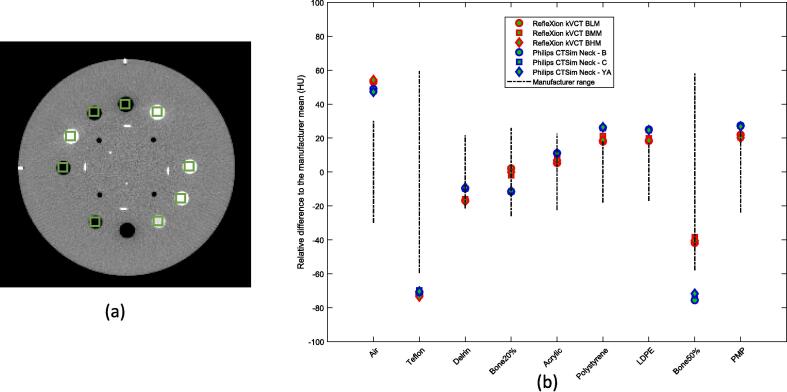
(a) An axial image of the HU module. (b) The mean HU measured for each insert indicated by the green ROI in (a) compared to the mean value and range from phantom manufacturer for RFX1 kVCT and CT simulator with default clinical protocols. (For interpretation of the references to colour in this figure legend, the reader is referred to the web version of this article.)

The mean and the standard deviation of the CT number of various inserts in the CTP682 module were measured on the images by a 6 × 6 pixel square ROI placed at the center of each insert as shown in Fig. 3(a).

##### 2.2.1.6 Geometrical accuracy

The geometrical accuracy was assessed by the measured distance between four 3 mm diameter holes that are positioned 50 mm apart in the CTP682 module as shown in Fig. S3 in supplementary material.

#### 2.2.2 Patient image quality

Examples of real patient images (informed consent was obtained) acquired in RFX1 kVCT prior to radiation treatments were presented and compared to Philips CT simulator images of the initial CT simulation for qualitative evaluation. Each patient was positioned in the same immobilization device for simulation and treatment and all images were acquired with free breathing. Default clinical protocols were used in both imaging systems.

### 2.3 Imaging dose measurement

The imaging dose for various protocols were measured using a 10 cm pencil ion chamber (Raysafe X2 CT Sensor, Fluke Biomedical, Everett, WA) and the computed tomography dose index (*CTDI*) phantom following the national standard method [17]. Three repeated measurements were made in the holes of the CTDI phantom at the central and 12 o’clock position. The CTDI_vol_ was computed as a weighted average by 1/3 of central and 2/3 of peripheral measurements and then divided by the pitch. The default kVp and mAs in clinical mode (Table 1) were used and the measured values were compared to the values reported by the vendor.

## 3 Results

### 3.1 RFX1 kVCT image quality evaluation and comparisons to Philips CT simulator

#### 3.1.1 Catphan

Fig. 1(b) shows the comparisons of the bar patterns (the zoomed-in square region in the original Catphan image in Fig. 1(a)). The bar pattern which represented a spatial resolution of 0.6 lp/mm can be identified in RFX1 CT for all three protocols. For CT simulator, the same bar pattern may be discernable by using the “YA” kernel. As shown in Fig. 1(c) this finding is consistent with the calculated MTF. The MTFs for the RFX1 kVCT are almost on top of each other but vary significantly for the CT simulator. The spatial frequency at 10% MTF is 0.68 (BLM)/0.67(BMM)/0.69(BHM) and 0.50(B)/0.53(C)/0.65(YA) for different protocols on RFX1 kVCT and CT simulator with different kernels, respectively, and that at 50% MTF is 0.36(BLM)/0.35(BMM)/0.35(BHM) and 0.30(B)/0.34(C)/0.45(YA).

The spatial frequency at 10% of MTF (“vanishing resolution”) is about 0.68 lp/mm for the RFX1 CT with all three protocols and about 0.65 lp/mm for the CT simulator with the sharpest reconstruction kernel “YA”. However, the 50% of MTF is significantly higher for CT simulator images with the “YA” kernel.

The cross-plane resolution represented by SSP are shown in Fig. 1(e) and (f). The FWHM on the SSP are 1.93 (BLM)/1.89(BMM)/1.88(BHM) mm for various protocols of RFX1 kVCT with nominal slice thickness of 1.25 mm and 2.97(B)/2.95(C)/2.89(YA) on CT simulator images with different reconstruction kernels with nominal slice thickness of 3 mm, respectively.

The spatial frequencies at 10% and 50% MTF as well as FWHM on the SSP for RFX1 kVCT and CT simulator are listed in Tables S1 and S2 in supplementary material.

Fig. 2(a) shows the axial images of the uniformity module of the Catphan604. The calculated image uniformity are 1.9(BLM)/1.7(BMM)/1.2(BHM) for three protocols of RFX1 kVCT and 1.1(B)/1.1(C)/1.0(YA) for CT simulator images with different kernels. The noise levels are shown at the bottom of each image in Fig. 2(a). Overall, the uniformity is better in CT simulator images than the RFX1 CT images. With default clinical imaging protocols (imaging dose not matched), the RFX1 kVCT images with high dose mode and CT simulator images reconstructed with kernels “B” and “C” have less noise. The noise level is lowest in CT simulator images with kernel “B” and highest in RFX1 kVCT images with low dose acquisition. After accounting for differences in slice thickness and imaging dose, the scaled noise on the RFX1 kVCT images are 8.3(BLM)/8.0(BMM)/8.7(BHM) which is higher than the CT simulator images except that with “YA” kernel.

As indicated by the orange arrow in Fig. 2(b), the diameter of the smallest identifiable disk in the group of nominal contrast of 1% on the RFX1 kVCT images are 7 mm, 5 mm, and 5 mm for low dose, medium dose, and high dose acquisition mode respectively. In contrast, the diameter of the minimal discernable low contrast disk on the CT simulator images is 7 mm for all the kernels “B”, “C”, and “YA”.

The CNR are shown at the bottom of each image in Fig. 2(b). The scaled CNR for the three RFX1 kVCT protocols are 1.2(BLM), 1.1(BMM), and 1.2(BHM) respectively. The CNR and scaled CNR measured for the low contrast insert are generally higher on the CT simulator images except on those with the “YA” kernel. The image uniformity, noise/scaled noise, CNR/scaled CNR for RFX1 kVCT and CT simulator images are also listed in Table S3 and S4 in supplementary material.

The mean CT numbers (shown in Fig. 3(b)) are very similar among three protocols for RFX1 kVCT as well as Philips CT simulator images with three reconstruction kernels. The agreement to the phantom manufacturer range is comparable between the RFX1 kVCT and CT simulator. The CT number for three media (air, Teflon, and Bone50%) on CT simulator and two (air and Teflon) on RFX1 kVCT deviates but is within 25 HU from the manufacturer range.

The horizontal and vertical distance between the four rods agree within 0.5 mm with the reference value of 50 mm for all of the modalities and protocols.

#### 3.1.2 Patient images

**Fig. 4:**
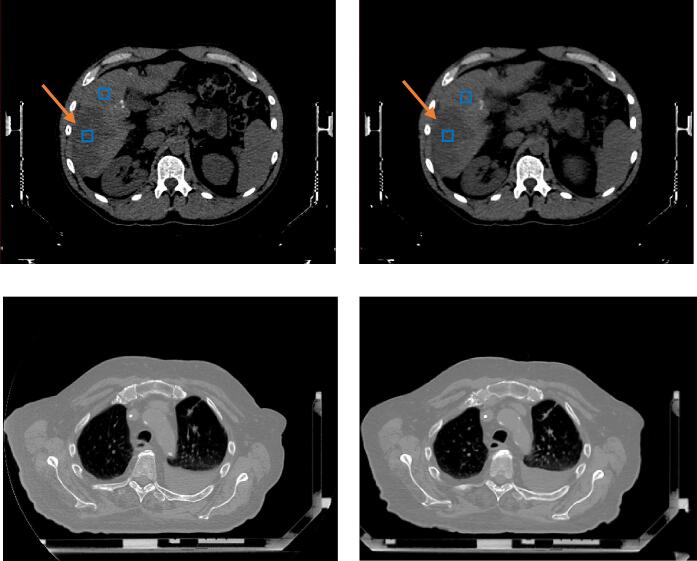
Patient images. Upper row: Liver SBRT, Lower row: Lung SBRT. Left column: RFX1 kVCT, Right column: CT simulator. The orange arrow points to a large low contrast lesion in liver. The noise and contrast were measured within the blue square ROIs. Display window is the same for each patient’s images. (For interpretation of the references to colour in this figure legend, the reader is referred to the web version of this article.)

We have treated seven SBRT patients since the commissioning of the RFX1 system. Images of a liver SBRT and a lung SBRT patients are shown in Fig. 4. The imaging protocol for RFX1 kVCT is body/high dose/slow couch with *CTDI_vol_* = 48.9 mGy and SBRT abdomen/lung protocols for CT simulator with kernel C (*CTDI_vol_* = 18.0 mGy for abdomen and 19.7 mGy for lung). As shown in the top row in Fig. 4, the visibility of the low contrast liver lesion is similar but the CT simulator images appear less noisy. To confirm this observation, square ROIs are placed in the lesion as well as in the normal liver (as background) to measure the noise/scaled noise and CNR/scaled CNR averaged from the nearest five slices. The noise / scaled noise is 20.5 /26.8 HU in RFX1 CT and 10.7/10.7 HU in CT simulator images, respectively; the CNR / scaled CNR is 1.1/0.85 in RFX1 CT and 2.3/2.3 in CT simulator images, respectively.

As shown in the bottom row in Fig. 4, the RFX1 CT images shows higher spatial resolution and is noisier than the CT simulator images. For patient images, the spatial resolution of the CT sim images is further degraded due to a larger pixel size of 1.37 mm.

### 3.2 Radiation dose measurement

The imaging dose measured in the *CTDI* phantom for the head/medium dose/medium couch (HMM) and body/medium dose/medium couch (BMM) protocols are 57.5 and 23.0 mGy respectively, which differ by 11.3% and 8.9% from the manufacturer’s specification. The coefficients of variation of the three repeated measurements range from 0.0% to 2.8%.

## 4 Discussion

In this paper, we presented the first comprehensive evaluation of image quality for the kVCT system in RefleXion X1 using Catphan604 images and two patient images. Physical phantom images and patient images were acquired with clinical protocols in RFX1 kVCT as listed in Table 1. Standard image quality metrics were extracted from various modules in the Catphan images for quantitative evaluation following the AAPM guidelines [18]. Although patient images were assessed mainly in a qualitative manner, noise and CNR for the liver SBRT patient were computed due to relative uniform HU in liver.

As summarized in Table 2, major imaging metrics were within vendor recommended specifications. The data presented in this paper also provides baseline image quality performance for the RFX1 kVCT system for our institution in establishing routine quality assurance program. The stability of the imaging performance will be studied later once long-term data is available.

**Table 2:** Summary of major image metrics on the RFX1 kVCT and vendor specifications. BMM = Body/Medium dose/Medium pitch; HMM = Head/Medium dose/Medium pitch.

	**RFX1 kVCT Test results**	**Vendor Specifications**
**MTF_50%_ (lp/cm)**	3.5	≥ 3.5
**Spatial resolution (lp/cm)**	6	≥ 4
**Contrast**	5 mm for BMM	5 mm object of 1% contrast
***CTDI_vol_* (cGy)**	2.3 (BMM)	≤ 2.8 (BMM)
	5.8 (HMM)	≤ 6 (HMM)
**Noise (HU)**	< 10	< 20
**Uniforimity (HU)**	< 2	< 25

Imaging metrics measured in the Catphan phantom images obtained in RFX1 kVCT were evaluated based upon a direct comparison to those measured on the reference Philips Brilliance Big Bore CT simulator images (scanning parameters listed in Table 1). Note that the *CTDI_vol_* reported in the RefleXion kVCT protocols are generally higher than that for the Philips CT simulator. The measured *CTDI_vol_* for two of the RFX1 kVCT protocols matched with nominal value within 12%. Ideally, the comparison of the imaging performance between two systems should be under the condition of the same imaging dose and reconstruction parameters. In the current study, the image quality of the CT simulator images was only used as a reference for evaluating those of the RFX1 kVCT images. We are fully aware of the differences in data acquisition and reconstruction between the two imaging systems. Thus, in comparing the noise and CNR, we also computed a scaled noise value by the square root of the imaging dose and slice thickness assuming only quantum noise was present in the reconstructed images. A separate study will be conducted to compare the imaging performance between the RFX1 kVCT and CT simulator by matching the imaging dose between the two systems.

Overall, higher spatial resolution, higher noise, better/comparable low contrast visibility and lower contrast-to-noise ratio were observed on the RFX1 kVCT images than the Philips CT simulator images using clinical protocol on both systems. The low contrast visibility appears higher in the Catphan images but are comparable for the patient images. A better agreement between the nominal slice thickness and FWHM of the SSP was observed for CT simulator because the reconstruction slice thickness was much larger than the detector width. For patient images, additional blur was introduced in CT simulator images due to a larger reconstruction FOV (70 cm) in the clinical protocol. These image quality differences may be attributed to the difference in image reconstruction algorithm (FDK versus iDose^4^) as well as the hardware used for data acquisition. The CT number accuracy and spatial linearity was comparable between the two imaging systems. The CT systems are usually calibrated using a water phantom, thus the CT number for a few density inserts in the Catphan phantom deviated from the manufacturer range for both imaging systems, “which is not unusual” according to the manufacturer manual of Catphan604 [19]. However, the maximum deviation is 25 HU. The CT number in our routine quality assurance for CT simulator with the Philips phantom showed no deviation from the manufacturer specified range, and the CT number to relative electron density calibration (HU-RED) was performed with the Gammex phantom (Sun Nuclear, Melbourne, FL) for dose calculation in the treatment planning system. Regarding noise performance, it may be further improved in RFX1 kVCT by implementing different image reconstruction kernels and iterative image reconstruction algorithms [20].

The imaging performance evaluation presented in this paper on RFX1 kVCT, including a direct comparison to the CT simulator images acquired with the default clinical protocols, demonstrated sufficient image quality for the purpose of IGRT. Similar low contrast visibility between the two imaging systems may lead to improved accuracy of image guidance based upon soft tissue contrast with RFX1 kVCT. With more accurate soft-tissue based patient setup, PTV margin may be further reduced [21], which will be explored later.

Although the main utilization of RFX1 kVCT is to provide 3D image guidance for radiotherapy, the comparable image quality between the RFX1 kVCT and CT simulator also sheds light on the possible extension of using the RFX1 kVCT system for advance applications such as on-line/off-line adaptive radiotherapy or as a backup CT system for patient simulation. For these applications, additional HU-RED calibration is required and further validation for organ segmentation and dose calculation accuracy need to be performed, and will be our future study.

In conclusion, we evaluated imaging performance of the kVCT in the RefleXion X1 system with both phantom images and patient images using clinical protocols on both systems. Major imaging metrics measured on the Catphan604 were within vendor-recommended tolerances. The comparable image quality between the RFX1 kVCT and Philips CT simulator may enable improved soft tissue-based image guidance and potential implementation of adaptive radiotherapy.

## Declaration of Competing Interest

The authors declare the following financial interests/personal relationships which may be considered as potential competing interests: The authors T. Zhuang, M-H. Lin, W. Lu and Bin Cai receive grants support from RefleXion Medical. A. Garant and T. Dan are PIs on two clinical trials funded by Reflexion Medical. R. Timmerman is on RefleXion Medical‘s scientific advisory board. No conflict of interests for other authors.
