# Supplementary material for: Evaluation of fan-beam kilovoltage computed tomography image quality on a novel biological-guided radiotherapy platform
Source: Phys Imaging Radiat Oncol. 2023 Apr 10;26:100438. doi: 10.1016/j.phro.2023.100438 (PMC10277913; doi:10.1016/j.phro.2023.100438)
Supplement: Supplementary data 1 [file mmc1.docx]

In this supplementary material, we present descriptions for the various modules of the Catphan604 phantom as well as three figures and four tables that are supplement to the evaluation of the kVCT image quality on a novel image-guided PET/CT linac, the RefleXion X1 system.

**Descriptions of Catphan604 phantom**

The CTP732 module contains 15 high contrast bars for spatial resolution evaluation as shown in Fig. 1(a). The CTP682 module contains multiple cylindrical inserts that allows for HU accuracy test (Fig. 3(a)). This module includes two pairs of wire ramping at a ${23}^{o}$ angle with respect to transverse direction that may be used for slice sensitivity profile measurement as shown in Fig. 1(d), and four holes that are 50 mm apart for spatial linearity assessment. The CTP729 module has a density within 2% of water and can be used to assess CT number uniformity and noise performance as shown in Fig. 2(a). The CTP730 module contains three groups of low contrast cylinders with nominal contrast of 0.3%, 0.5% and 1.0% and diameters from 2 mm to 15 mm as shown in Fig. 2(b).

**Supplementary figures and tables**

**Fig. S1** shows a cross-section view of the major components of the RefleXion X1 (RFX1) system. The kV imaging system in RFX1 consists of a kV x-ray tube which is capable of potentials from 80 to 140 kVp and operates at 120 kVp in clinic mode, with a 16-row detector. The kV source-to-imager-distance is 1133 mm and the source-to-axis distance is 643 mm. The kV imaging plane is 386 mm away from the MV isocenter. The kV source-detector assembly rotates at a maximum angular speed of one rotation per second.


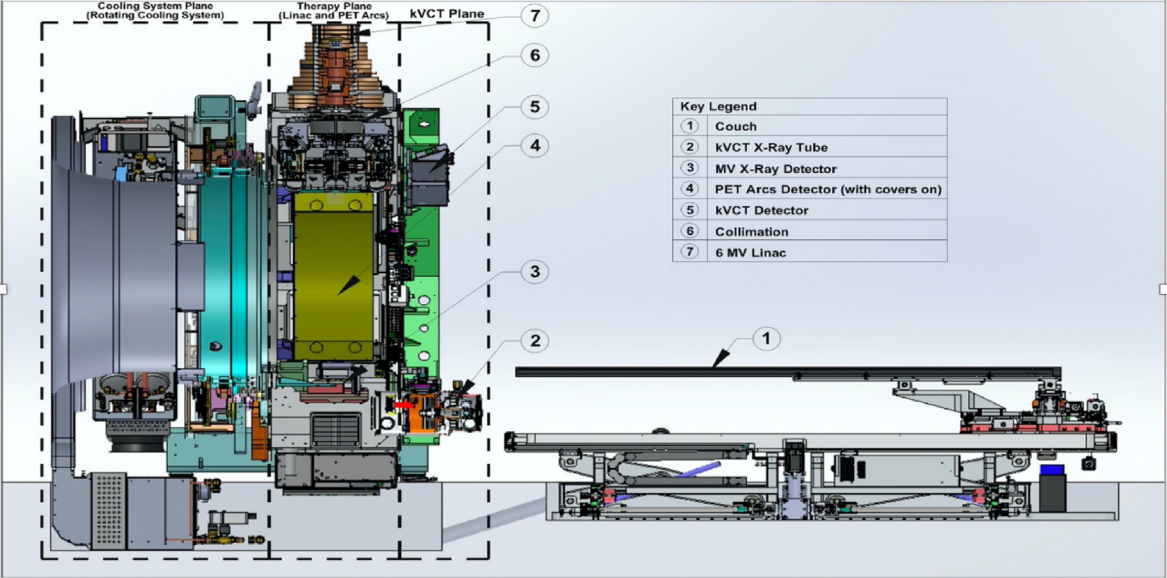


Fig.S1. Major components of the RefleXion X1 system including 6 MV Linac, kVCT and PET imaging systems[8].

**Fig. S2** shows the process of calculating MTF using a circular edge method on the axial image of the uniform module in Catphan604. We chose the circular edge from the uniformity module CTP729 to compute the MTF. In this study, we computed the pre-sampled MTF with an oversampling factor of five. As shown in **Fig. S2**, we first select a horizontal line passing through the center to compute the edge spread function (ESF) with a resolution of $\Delta\xi=\Delta x/5$ where $\Delta x$ is the image pixel size. For every point ξ on the line segment, the value for ESF($x$) is obtained as the average HU of the pixels that are on the circle (the center of the pixel is within half of $\Delta\xi$ from the circle) that contains this point. The line spread function (LSF) is computed as the derivative of the ESF and the MTF is obtained as 1D Fourier transform of the LSF. The MTF was averaged over five nearby slices.

Data rebinning

Distance (mm) Distance (mm) Spatial frequency (mm^-1^)

1. (b) (c) (d)

HU

Relative intensity

Relative intensity

Fig. S2. Demonstration of steps to compute the pre-sampled MTF. (a) The ESF is to be computed on the red dash-dotted line segment. (b) The value at a point $\xi$, indicated by the blue dot, is obtained by averaging the HU value of the pixels that are on the circle (red dashed line) passing through $\xi$ as shown in (a). (c) The LSF is computed as the derivative of the ESF. (d). The MTF is the 1D Fourier transform of LSF.

**Fig. S3** shows the method to evaluate the geometrical linearity.

Geometrical linearity

*Fig. S3. The distance between the two horizontal holes and two vertical holes connected by the orange dashed lines were calculated to assess the geometrical linearity.*

**Tables S1** and **S2** list the spatial frequencies at 10% and 50% MTF as well as FWHM on the SSP for RFX1 kVCT and CT simulator. **Tables S3** and **S4** list the image uniformity, noise/scaled noise, CNR/scaled CNR for RFX1 kVCT and CT simulator images.

Table S1. MTF at 50% and 10% for the two imaging systems.

|  | **RFX1** | | | **CT Sim** | | |
| --- | --- | --- | --- | --- | --- | --- |
|  | **BLM** | **BMM** | **BHM** | **B** | **C** | **YA** |
| **MTF_50%_ (mm^-1^)** | 0.36 | 0.35 | 0.35 | 0.30 | 0.34 | 0.45 |
| **MTF_10%_ (mm^-1^)** | 0.68 | 0.67 | 0.69 | 0.50 | 0.53 | 0.65 |

Table S2. FWHM on the SSP computed for RFX1 kVCT and CT simulator as compared to the nominal value.

|  | **RFX1** | | | **CT Sim** | | |
| --- | --- | --- | --- | --- | --- | --- |
|  | **BLM** | **BMM** | **BHM** | **B** | **C** | **YA** |
| **FWHM/ nominal Slice Thickness (mm)** | 1.93/1.25 | 1.89/1.25 | 1.88/1.25 | 2.97/3.00 | 2.95/3.00 | 2.89/3.00 |

Table S3. Statistics of noise level of the five ROIs in the uniformity module as well as the uniformity measured by the maximum HU differences between the peripheral and central RO for the two imaging systems.

|  | **RFX1** | | | **CT Sim** | | |
| --- | --- | --- | --- | --- | --- | --- |
|  | **BLM** | **BMM** | **BHM** | **B** | **C** | **YA** |
| ${\boldsymbol{\vert}\boldsymbol{ROI}_{\boldsymbol{peri}}\boldsymbol{-}\boldsymbol{ROI}_{\boldsymbol{ctr}}\boldsymbol{\vert}\text{ }}_{\boldsymbol{Max}}$ | 1.9 | 1.7 | 1.2 | 1.1 | 1.1 | 1.0 |
| **Noise** | 9.9 ± 1.8 | 7.9 ± 1.5 | 6.0 ± 1.1 | 4.9 ± 0.5 | 5.9 ± 0.5 | 9.5 ± 0.8 |
| **Scaled noise** | 8.3 ± 1.5 | 8.0 ± 1.5 | 8.7 ± 1.5 | 4.9 ± 0.5 | 5.9 ± 0.5 | 9.5 ± 0.8 |

Table S4. The CNR measured using the 15mm low contrast insert (1% contrast).

|  | **RFX1** | | | **CT Sim** | | |
| --- | --- | --- | --- | --- | --- | --- |
|  | **BLM** | **BMM** | **BHM** | **B** | **C** | **YA** |
| **CNR** | 1.0 ± 0.2 | 1.1 ± 0.3 | 1.7 ± 0.3 | 2.4 ± 0.6 | 2.0 ± 0.5 | 1.2 ± 0.3 |
| $\hat{\boldsymbol{CNR}}$ | 1.2 ± 0.3 | 1.1 ± 0.3 | 1.2± 0.2 | 2.4 ± 0.6 | 2.0 ± 0.5 | 1.2 ± 0.3 |
